# Tracing Developmental Trajectories of Oppositional Defiant Behaviors in Preschool Children

**DOI:** 10.1371/journal.pone.0101089

**Published:** 2014-06-27

**Authors:** Lourdes Ezpeleta, Roser Granero, Núria de la Osa, José Blas Navarro, Eva Penelo, Josep M. Domènech

**Affiliations:** 1 Unitat d’Epidemiologia i de Diagnòstic en Psicopatologia del Desenvolupament, Departament de Psicologia Clínica i de la Salut, Universitat Autònoma de Barcelona, Barcelona, Spain; 2 Departament de Psicobiologia i Metodologia de les Ciències de la Salut, Universitat Autònoma de Barcelona, Barcelona, Spain; University Children's Hospital Tuebingen, Germany

## Abstract

***Objective*** Previous studies on developmental trajectories have used ad hoc definitions of oppositional defiant behaviors (ODB), which makes it difficult to compare results. This article defines developmental trajectories of ODB from ages 3–5 based on five different standard measurements derived from three separate instruments.

**Method:**

A sample of 622 three-year-old preschoolers, followed up at ages 4, 5, and 6, was assessed with the five measures of oppositionality answered by parents and teachers. Growth-Mixture-Modeling (GMM) estimated separate developmental trajectories for each ODB measure for ages 3 to 5.

**Results:**

The number of classes-trajectories obtained in each GMM depended on the ODB measure, but two clear patterns emerged: four trajectories (persistent low, decreasers, increasers/high increasers, persistent moderate/persistent high) or three trajectories (persistent low, decreasers, increasers/high increasers). Persistent high trajectories accounted for 4.4%–9.5% of the children. The trajectories emerging from the different ODB measures at ages 3 to 5 discriminated disruptive disorders, comorbidity, use of services, and impairment at age 6, and globally showed a similar pattern, summarizing longitudinal information on oppositionality in preschool children in a similar way.

**Conclusions:**

Trajectories resulting from standard scales of the questionnaires have predictive validity for identifying relevant clinical outcomes, but are measure-specific. The results contribute to knowledge about the development of ODB in preschool children.

## Introduction

The recognition that some disorders have their roots in the preschool period and that these problems are stable and continue into later life [1] has recently led to substantial growth in the study of psychopathology among pre-schoolers. Oppositional defiant disorder (ODD) is among the most prevalent disorders in this age range [2], [3]. The varied concurrent and successive comorbidity shown by ODD [4], along with its associated problems in terms of daily functioning in different contexts [5], highlight the importance of efforts to understand and prevent this disorder.

The study of developmental trajectories enables us to understand the evolution of disorders, with regard to their level and to their growth or decline over time [6]. Such study helps researchers identify the causes and consequences of the behaviors traced. In this sense, the identification of trajectories constitutes a highly valuable strategy for our knowledge of psychopathologies, their associated factors and the predictive associations between them. The results of these types of studies are also useful for identifying groups of children with different needs over the course of their development, and for designing proper therapeutic and preventive interventions. The preschool age is when interventions may be most effective [7], and it is therefore important to study this developmental period closely.

Data for ODD developmental trajectories from early preschool ages are scarce. Miner and Clarke-Stewart [8] studied the normative trajectories of externalizing behaviors in a sample of the general population from ages 2 to 9, which was assessed five times with a short externalizing behavior scale from the Child Behavior Checklist (CBCL) [9], [10], filled out by mothers and teachers. There was a decline in externalizing behavior from 2 to 9 years old, which was most pronounced from 2 to 7 and then became more gradual. It is also of note that mothers rated children higher than teachers did. Peticlerc et al. [11] used three items of the CBCL/1½-5 that were indicative of disregard for rules (defiant, not guilty, insensitive to punishment), an important dimension of oppositional defiant and conduct disorder, and found four trajectories in a sample of 1,942 children aged 29 months to 74 months: very low (9.1%), low (56.9%), moderate (29.7%), and chronic (4.3%). The trajectories were stable and reflected different degrees of severity. Males, maternal antisocial behavior, and postpartum depression in a parent were most likely to be associated with a chronic trajectory. In a sample of boys from low-income families, tracked from 1.5 to 10 years of age, Shaw et al. [12] found four trajectories of conduct problems, defined through five items of the CBCL (cruelty to animals, disobedience, fights, physical attacks, temper tantrums), as answered by the mothers: low (10.1%), moderate desisters (33.2%), medium decline (49.9%) and chronic (6.7%). Children in the chronic group scored high in fearlessness, and their mothers were younger, more depressed and more child-rejecting. All of these studies were based on the modification of existing measures and produced different definitions, which impedes comparisons.

Previous work on developmental trajectories has contributed to an understanding of the externalizing disorders, but it also has some shortcomings. First, some of these studies have been overly focused on boys and on samples from low socioeconomic levels, which limits the generalizability of results. Trajectories have also mostly been derived based on just one informant (the mothers) and one instrument, and the use of varying and idiosyncratic definitions also makes it difficult to compare results across studies. Furthermore, there are concerns that diagnostic instruments and screening/scale scores do not tap the same phenomena, since screens are only modestly associated with diagnosis. Therefore, it is worthwhile finding out whether the same trajectories emerge when ODD symptoms based on a diagnostic interview are assessed, as opposed to scale scores. In the emerging field of preschool psychopathology there is a need for cross-instrument data on developmental trajectories, as traced using different assessment tools, not only for consolidating the field, but also with a view to providing appropriate estimations of the evolution of disorders in this segment of the population. In this way it may be possible to improve service provision. The aim of this study is to contribute data on the characteristics of developmental trajectories of oppositional defiant behaviors (ODB) obtained with three of the most widely-used instruments for the assessment of this disorder (DSM-IV diagnostic interview, CBCL and Strengths and Difficulties Questionnaire -SDQ) in a sample of Spanish three-year-olds in their first year of preschool education. There were two specific objectives: 1) to trace the developmental trajectories of ODB from ages 3 to 5, based on different instruments and using the scales that clinicians and researchers most typically employ; and 2) to ascertain the discriminative ability of these trajectories for different outcomes at age 6 in order to evaluate the usefulness of each instrument for this purpose. The results of this study may help clinicians and researchers interested in the evolution of ODB at these ages to choose the most appropriate instrument according to their goals, which might in turn improve identification of the needs of affected children.

## Method

### Participants

The data analyzed in this work come from a longitudinal study on psychopathological risk factors from age 3 described in [3] (Figure S1 online). The sample design involved a two-phase sampling. In the first phase a random group of 2,283 families obtained from the census of 3-year-old preschoolers in Barcelona (Spain) were contacted and invited to participate. In total, 1,341 families (58.7%) agreed to take part (33.6% of high socioeconomic status, 43.1% middle and 23.3% low; 50.9% were boys). To ensure the participation of children with possible behavioral problems, the first phase sampling applied the parent-rated SDQ^3–4^ conduct problems scale [13] plus four ODD DSM-IV-TR symptoms, which were used as screening for ODB problems. Two groups were potentially considered: screen positive (all children with SDQ^3–4^ scores ≥4, Percentile 90, or with a positive response for any of the 8 DSM-IV ODD symptoms) and screen negative (a random group comprising 28% of children who did not reach the positive threshold). The number of refusals in this stage of the sampling was *n* = 135 (10.6%), and these children did not differ in sex (*p* = .815) or type of school (*p* = .850) from those who did agree to participate (the only difference was in SES, with higher participation ratio for high socioeconomic levels, 86.2% *vs*. 73.6%; *p* = .007).

The second sampling phase involved the n* = *622 children (417 from the positive screen group −49.4% boys− and 205 from the negative screen −51.2% boys), who were selected for the follow-up. The first complete assessment of this sample was at age 3. Demographic characteristics are shown in Table 1 (and Table S1 online). At age 4,603 children remained in the follow-up (303 boys), at age 5 there were 570 (288 boys), and at age 6 there were 511 (256 boys). No differences in sex (χ^2^ = 1.39, df = 1, *p* = .29), SES (χ^2^ = 5.27, df = 2, *p* = .08) or type of school (χ^2^ = 0.51, df = 1, *p* = .53) were found on comparing completers and drop-outs.

**Table 1:** Demographic Characteristics of the Sample (*N* = 622).

Age (mean; *SD*)		3.8	(0.33)
Sex (*N*;%)	Male	311	(50.0)
Race/ethnicity (*N*;%)	Non-Hispanic white	557	(89.5)
	Hispanic-American	46	(7.4)
	Other	19	(3.1)
Family socioeconomic status (*N*;%)	High	205	(33.0)
	Mean-high	280	(45.0)
	Low	137	(22.0)

### Measures

The *Diagnostic Interview of Children and Adolescents for Parents of Preschool Children* (DICA-PPC [14]) is a semi-structured interview for parents of children aged 3 to 7 that follows the DSM-IV-TR criteria [15]. The schedule is interviewer-based, as it is the interviewer who decides about the presence/absence of the symptoms, considering the answers provided by the parents and the definitions of the symptoms in the manual. The characteristics of the symptoms, the methods of identifying those characteristics and how to code the symptoms were taught during the training. An intensive training period lasting one week included an overview of the developmental psychology and psychopathology of preschool children, as well as interviewing skills. Subsequently, a longer training period was made up of 4 phases: 1) Study of the interview schedule; 2) Practice interviews and role-playing in simulated interviews; 3) Listening to and coding of audio-recorded real interviews; and 4) Observation and coding of live interviews. The criterion for being ready for the field was to obtain a mean agreement with an expert kappa ≥0.80 for all the questions in at least five interviews. The team of interviewers consisted of two Ph.D. clinical psychologists, three psychologists with masters degrees and five psychology students.

Inter-interviewer agreement was examined in a pilot study with 13 interviews involving children from public pediatric primary care, whose families accepted to participate. Mean age of the children was 5.54 years (SD  =  0.97). For each interview the kappa coefficient [16] between the interviewer and each of the observers (raters) was calculated for all the interview questions and for the diagnoses. The interviews were recorded and then rated by a team of five interviewers, resulting in 65 observations. All the interviewers took the role of “interviewer” and “raters”. Kappa coefficients of the rating of all the interview questions ranged from.64 to.94 (mean kappa.78, 95% CI:.73 to.83) indicating good to excellent agreement between interviewers. The agreement was significant and very good for disruptive behavior disorders (κ = .91).

Presence/absence of the eight ODD symptoms was used to derive trajectories. The diagnoses analyzed as outcomes were disruptive behavior disorders (ADHD, ODD and CD), depressive disorders (major depression, dysthymia and minor depression) and anxiety disorders (separation and generalized anxiety, specific and social phobia), in addition to the number of CD-aggressive symptoms (bullying, fighting, weapon use, cruelty to people, cruelty to animals, stealing with confrontation, and forced sex) and CD-non-aggressive symptoms (fire-raising, vandalism, breaking and entering, lying, and stealing without confrontation). Comorbidity was defined as the presence of more than one disorder among those assessed in the diagnostic interview. Use of services was registered after assessment of the symptoms of each disorder. The interviewer recorded whether a professional had been consulted about the symptoms and whether the child received any treatment for the symptoms (psychological, pharmacological or other).

The *Child Behavior Checklist* (CBCL/1½-5 [17]) measures behavioral and emotional problems according to parents' perception. The aggressive behavior empirical scale (18 items; *not true*, *somewhat true*, *very true*) and the DSM-scale of oppositional defiant problems (5 items) were used to derive the oppositional defiant behaviors. Cronbach's alpha of the scales in the sample was, respectively, .84 and .74.

The *Strengths and Difficulties Questionnaire* (SDQ [13]) is a brief screening questionnaire for the mental health of children. The conduct problems scale (5 items; *not true*, *somewhat true*, *certainly true*) was completed by parents and teachers. Cronbach's alpha in the sample was, respectively, .59 and .73.

The *Children's Global Assessment Scale* (CGAS [18]) is a global measure of functional impairment rated by the interviewer based on information from the diagnostic interview. Scale scores range from 1 (maximum impairment) to 100 (normal functioning). Scores above 70 indicate normal adaptation.

All the measures were administered yearly. Table S4 online shows the items in each scale of the instruments used for the trajectories, as well as the item scores and the scale's theoretical range.

### Procedure

The project was approved by the ethics review committee of the authors' institution (Comissió d’Etica en l’Experimentació Animal I Humana). Informed written consent was obtained from parents of the children participating in the study, as approved by the ethics committee, in which the confidentiality of data was guaranteed. In Spain, preschool education is financed from public funds, and 3-year-olds are in preschool education. Families were recruited at the schools and also gave written consent. All families of children from P3 (3-year-olds) in the participating schools were invited to answer the SDQ^3-4^. Families who agreed and met the screening criteria were contacted by telephone and interviewed at the school for each assessment. The interviewer team, which included two clinical psychologists with Ph.D., two psychologists with masters degrees and psychology students, was specifically trained, and all interviewers were blind to screening group (see [14]). All interviews were audio-recorded and supervised. After the interview, the interviewer completed the CGAS, and the teacher's SDQ was given out for completion before the end of the academic year. The data were collected once a year between November 2009 and July 2013, with an average interval between the first and second assessment of 11.01 months (SD = 1.15) and 12.45 months (SD = 1.19) between the second and third assessment. Average interval between parent-family assessment and teacher's report in the follow-ups was 1.41 months (SD = 1.79).

### Statistical Analysis

The trajectories were obtained through Growth-Mixture-Modeling (GMM) in MPlus7 using the sampling weight procedure within the Variable command to account for the multi-sampling design (each child was weighted by the inverse proportion to the probability of selection in the second phase of the sampling), defining the Robust Maximum Likelihood (MLR) estimator in the Analysis command (a full-information method [19], [20]) and using Lo-Mendell-Rubin [21] as a measure to determine the number of classes. Five individual GMMs estimated separate developmental trajectories for each ODB measure (DSM-IV-ODD symptoms, CBCL-behavior scale, CBCL-DSM-ODB scale, SDQ-Parents conduct scale and SDQ-Teachers conduct scale) registered for the children's ages: 3-4-5 years old. Selection of the number of trajectories for each of the five measures considered was based on [22]: a) the lowest Bayesian information criterion (BIC) for the model (compared with other solutions); b) entropy (measure of the model's discriminative capacity for classifying children, that is, its ability to identify individuals following the different trajectories) above .80; c) high on-diagonal average values (around .80) in the matrix containing the probabilities of membership (that is, high average latent class probabilities for most likely latent class membership by latent class); d) no less than 4% of participants in a class/trajectory (to allow statistical comparisons); and e) the best clinical interpretability.

The discriminant capacity of trajectories on psychopathology and functioning at age 6 was estimated with the SPSS20-Complex Samples (CS) module. A design was drawn up according to the multi-stage sampling, assigning to each child a weight equal to the inverse proportion of the probability of selection in the second phase of the sampling. Logistic regression models (CSLOGISTIC procedure) were obtained for binary outcomes and General Linear Models (CSGLM procedure) were applied to quantitative criteria, both defining Robust estimation. These analyses were adjusted for the presence of comorbidities other than those included in the models at baseline (age 3 years) to obtain the specific discriminant capacity of the trajectories. Predictive capacity for the trajectories was estimated through the change in R^2^ (ΔR^2^), comparing the second step-block including the trajectories with the previous step-block including only the covariate “other comorbidity”

## Results

**Figure 1:**
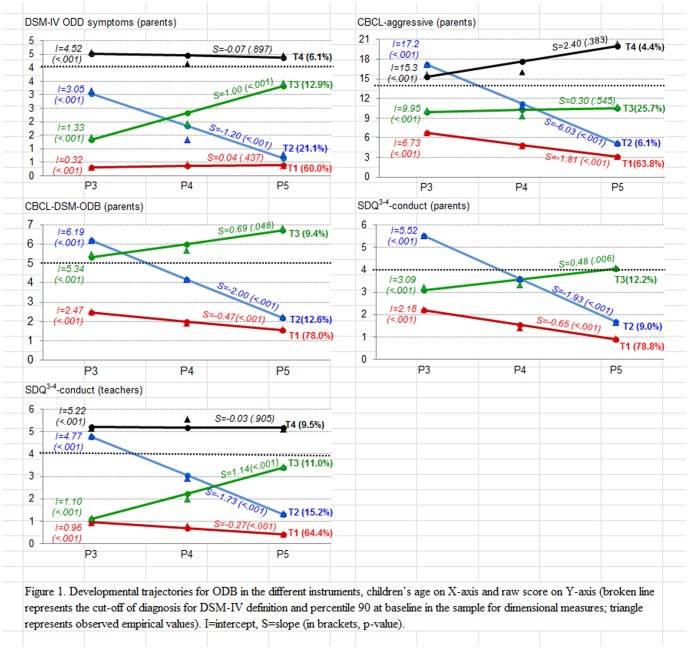
Developmental trajectories for ODB in the different instruments, children's age on X-axis and raw score on Y-axis (broken line represents the cut-off of diagnosis for DSM-IV definition and percentile 90 at baseline in the sample for dimensional measures; triangle represents observed empirical values). *S = slope (in brackets, p-value)*.

Table S2, online, shows the goodness-of-fit for the GMM analyses. Different candidate models were considered for each measure with a number of trajectories/groups ranging from one to four (more groups were not considered due to the lack of convergence and/or very small classes). The final models selected yielded the lowest BIC indices (except for the parent-rated SDQ-conduct and CBCL/1½-5 DSM-ODB, but the three-group models were retained as the final solutions because fit criteria were not obtained for solutions with larger numbers of classes-trajectories), good entropy, high on-diagonal values in the matrix with the average latent class probabilities, and adequate sample size and clinical interpretability. Figure 1 shows the final model selected for each measure. No statistical differences between trajectories were found for sex or SES.

### Trajectories derived from DSM-IV-ODD symptoms reported by parents

The results of the GMM identified four trajectories of ODD symptoms. Trajectory 1 (*N* = 373, 60.0%) represented those children with a persistently low number of ODD symptoms from ages 3 to 5 (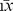 = 0.32, 0.42 and 0.39); trajectory 2 (*N* = 131, 21.1%) represented decreasers in ODD symptoms, starting at age 3 with a high mean number of symptoms (3.1) and with a low mean by age 5 (0.65); trajectory 3 (*N* = 80, 12.9%) represented increasers, that is, children starting with 1.33 mean symptoms at age 3, and showing an increase in mean number of symptoms to 3.32 at age 5; and trajectory 4 (*N* = 38, 6.1%) represented those with a persistently high number of symptoms (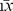 = 4.52, 4.45 and 4.37); this group had a mean number of symptoms above the DSM-IV diagnostic threshold across the three assessments.

### Trajectories derived from parents' CBCL/1½-5 empirical and DSM scales

For the CBCL-aggressive behavior scale, four trajectories were identified. Four children were excluded from the GMM due to missing-values in the three follow-ups for this ODB measure. Trajectory 1 (*N* = 394, 63.8%) represented persistently low mean scores on the scale from ages 3 to 5 (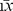 = 6.73, 4.92 and 3.11); trajectory 2 (*N* = 38, 6.1%) represented decreasers in aggressive behavior scores who started with a high mean score (17.2) at age 3 and had lower means by ages 4 (11.2) and 5 (5.13); trajectory 3 (*N* = 159, 25.7%) represented persistently moderate scores (mean scores 9.95, 10.2 and 10.5 at ages 3, 4, and 5, respectively); and trajectory 4 (*N* = 27, 4.4%) represented the high increasers (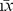 = 15.3, 17.7, 20.1), that is, children who remained at high scores above cut-off in the three assessments, and even showed increases in these scores.

For the CBCL DSM-ODB scale, three trajectories were identified. Five children were excluded from the GMM due to missing values in the three follow-ups for this ODB measure. Trajectory 1 (*N* = 481, 78.0%) represented persistently low scores on the DSM-ODB scale from ages 3 to 5 (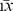 = 2.47, 2.00, 1.54); trajectory 2 (*N* = 78, 12.6%) represented decreasers with mean scores of 6.19, 4.19 and 2.19 at ages 3, 4 and 5, respectively; and trajectory 3 (*N* = 58, 9.4%) represented high increasers, that is, children with a high mean number of symptoms at age 3 (5.34), and who showed an increase in mean score at ages 4 (6.02) and 5 (6.71), also remaining above cut-off at all three assessment points.

### Trajectories derived from the parent- and teacher-reported SDQ conduct scale

For the conduct scale of the SDQ completed by parents, three trajectories emerged. Trajectory 1 (*N* = 490, 78.8%) represented persistently low scores on the conduct scale from ages 3 to 5 (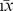 = 2.18, 1.53, 0.89); trajectory 2 (*N* = 56, 9.0%) represented decreasers in conduct scale scores with means of 5.52, 3.60 and 1.67 for ages 3, 4 and 5, respectively; and trajectory 3 (*N* = 76, 12.2%) represented increasers, that is, children starting with a mean score of 3.09 at age 3, and whose mean score had increased by ages 4 (3.56) and 5 (4.04).

For the SDQ conduct scale answered by teachers, four trajectories were identified. Two children were excluded from the GMM due to missing values in the three follow-ups for this ODB measure. Trajectory 1 (*N* = 399, 64.4%) represented persistently low scores between ages 3 and 5 on the conduct scale (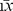 = 0.96, 0.69, 0.42); trajectory 2 (*N* = 94, 15.2%) represented decreasers in conduct scale scores with means from 4.77, 3.05 and 1.32 for ages 3, 4 and 5, respectively; trajectory 3 (*N* = 68, 11.0%) represented increasers, that is, children starting with a mean score for symptoms at age 3 of 1.10 and whose mean score had increased by ages 4 (2.24) and 5 (3.39); and trajectory 4 (*N* = 59, 9.5%) represented persistently high scores, these children maintaining a high mean score above cut-off at ages 3, 4, and 5 (5.22, 5.19, and 5.16, respectively).

### Agreement between trajectories

The kappa (κ) and the global agreement (relative observed concordance between classes) was estimated for similar trajectories yielded by the different instruments: one concordance matrix was calculated for the 4-class trajectories solutions (DSM-IV-ODD, CBCL-aggressive and SDQ-conduct-parents) and another for the 3-class trajectories solutions (CBCL-DSM-ODB and SDQ-conduct-teachers). Agreement indexes were low: DSM-IV-ODD and CBCL-aggressive κ = .32 (65.9% global agreement), DSM-IV and SDQ parents κ = .24 (67.6% agreement), CBCL-aggressive and SDQ parents κ = .31 (72.1% agreement), and CBCL-DSM-ODB and SDQ teacher κ = .10 (61.4% agreement).

### Discriminant capacity of the trajectories

The shape and number of trajectories yielded by the parents' CBCL DSM-ODB were similar to those of the parents' SDQ-conduct; the parents' DSM-IV trajectories were similar to those of the teachers' SDQ-conduct; and the trajectory of the parents' CBCL-aggressive was the most distinctive. Table 2 summarizes the ability of this set of trajectories to discriminate DSM-IV diagnoses, comorbidity, use of services, functional impairment and the number of CD symptoms at age 6 (detailed information in Table S3, online). These results are adjusted for the presence of comorbidities other than those included in the model at baseline (i.e., for the prediction of ADHD at 6 years, ADHD, ODD, conduct disorder, depression and anxiety at 3 years were controlled for), to obtain the adjusted predictive capacity for the trajectories in relation to outcomes (ΔR^2^ values tabulated correspond to the change in predictive capacity between the first step entering the covariate “other comorbidities” and the second step adding the trajectories). All instruments yielded statistically significant predictions for disruptive disorders (ADHD and ODD) (except the DSM-IV, which did not significantly discriminate ADHD), depression, comorbidity, use of services and impairment. DSM-IV trajectories showed very good discriminative power (significant ΔR^2^ coefficients above 20%) for ODD and depression, and good predictive capacity (significant ΔR^2^ coefficients above 10%) for the group of disruptive disorders, impairment and comorbidity. CBCL-aggressive was the best measure for predicting the disruptive disorders group, ADHD, comorbidity and use of services. CBCL DSM-ODB and the parent SDQ showed good predictive capacity for disruptive disorders (particularly ODD) and depression. The teacher SDQ showed good predictive power for disruptive disorders (and particularly ADHD), and very good predictive power for depression. No significant predictor emerged for anxiety disorders at age 6. The only significant predictor of the number of CD aggressive symptoms at age 6 was teacher SDQ trajectories, but the effect size of this prediction was small. For the number of CD non-aggressive symptoms the significant predictors were CBCL- DSM-ODB and SDQ, but the effect sizes of ΔR^2^ values were low.

**Table 2:** Discriminative Ability of the Trajectories (columns) for outcomes (rows): Diagnoses, Comorbidity, Use of Services and Functional Impairment at Age 6 (ΔR^2^).

	DSM-IV ODD	CBCL- Aggressive	CBCL- ODB	SDQ Parent	SDQ Teacher
1Disruptive disorders	**.152**	**.223**	**.162**	**.136**	**.144**
1ADHD	.028	**.136**	**.044**	**.050**	**.129**
1ODD	**.247**	**.198**	**.182**	**.131**	**.074**
1Depression	**.272**	**.134**	**.101**	**.123**	**.207**
1 ^,^ 3Anxiety disorders	.002	.015	.010	.004	.012
1Comorbidity	**.096**	**.175**	**.056**	**.035**	**.065**
1Use of services	**.048**	**.099**	**.022**	**.034**	**.040**
^2^Impairment (CGAS: total)	**.152**	**.136**	**.058**	**.043**	**.033**
^2^# CD aggressive symptoms	.014	.016	.037	.019	**.038**
^2^# CD non-aggressive symptoms	.013	.025	**.020**	**.026**	**.021**

1 Logistic Regression for binary outcomes and ^2^General Linear Model for quantitative.

3 Includes separation-generalized anxiety, specific-social phobia.

Results adjusted for other comorbidities at baseline (age 3).

ΔR^2^: change in R^2^ when entering the trajectories in comparison with the previous step including only the covariate “other comorbidity”.

Bold: significant ΔR^2^ (p <.05).

## Discussion

This study identified several developmental trajectories of oppositional-disorder-related behavior from ages 3 to 5 for the different definitions of the instruments used. The trajectories identified showed good ability to discriminate disruptive disorders, depression, comorbidity, use of services and impairment at age 6. Goodness-of-fit, clinical interpretability and convergence supported the validity of the empirical trajectories.

The number of trajectories ranged between 3 (for CBCL-DSM-ODB and parents' SDQ) and 4 (for the DSM-IV, CBCL-aggressive and teachers' SDQ). The trajectories yielded by each instrument accounted for different children, as shown by the low agreement indexes. This finding suggests that the composition of trajectories is specific to each definition, and that the instruments are not measuring exactly the same construct, thus highlighting the advisability of using various instruments answered by different reporters in the assessment of children with oppositional defiant disorders. We did not find differences between trajectories for sex or SES, which is in line with the results of recent studies in preschoolers that found no association of SES with diagnosis [2] and no sex differences for trajectories [23], pointing out that sex differences emerge later in development.

A persistently low trajectory was always found, accounting for the majority of non-symptomatic children at age 3 who maintained this level until age 5 (range from 60% to 78%). Using standard definitions from a range of instruments, the parents and teachers in our study discriminated the oppositional behaviors as non-normative in the preschool age range.

For all instruments it was possible to identify a decreasers trajectory. In 6.1% to 21% of the children that start out with marked negativistic behavior, the developmental trajectory in these behaviors is a decreasing one. An increaser trajectory was identified for the DSM-IV and SDQ-conduct (parents and teachers). This trajectory accounts for 11% to 12.9% of cases. Finally, for the DSM-IV, CBCL-aggressive and SDQ-teachers, a stable high trajectory was found. The proportion of children in the high trajectory varied across the different instruments used, from 4.4% (CBCL-aggressive behavior) to 9.5% (SDQ-conduct teacher) (the parents' SDQ-conduct did not yield a chronic trajectory). Previous studies have shown the stability of disruptive behavior over long periods of time [24] and highlighted the usefulness of the study of developmental trajectories for focusing intervention and prevention initiatives. High and mean-level trajectories permit the identification of groups of children that should be the target of preventive efforts. Given the concurrent and successive comorbidity associated with ODD, preventive intervention oriented toward ODD may be successful for reducing not only disruptive disorders, but also the chain of disorders associated with ODD [25].

Although not for all the instruments, we were able to trace trajectories according to two informants: parents and teachers in the case of the SDQ conduct scale. Keiley et al. [26] reported that the trajectories of scores on the externalizing scale (Achenbach's instruments) from ages 5 to 12 were lower in the case of mothers and higher for teachers. Similarly, in our study, considering the SDQ answered by both parents and teachers, parents tended to report a decrease in conduct problems from ages 3 to 5, while teachers identified a group of children who maintained high scores in conduct problems for all three assessments. The discrepancies observed between parents and teachers might be attributable, in part, to differences in the context or situation in which the child's behavior is observed [27]. In accordance with Keiley et al. [26], we also observed that the development of conduct problems differs according to reporter or context. Recently, ODD has been conceptualized as a source-specific phenomenon: different groups of children, with different characteristics, are identified depending on the informant and on how the information is combined [28]. However, the characteristics of the informant (psychological problems, stress, socioeconomic status, perspective on the behavior, etc.), family status, and the characteristics of the child (problem type, non-clinical population) can also contribute to the discrepancies [29]. Therefore, for the study of the developmental trajectories of ODB it would seem advisable to have available information from several reporters. We also noted that trajectories were “measure-specific”, which has implications for research and clinical practice: the results depend on the instrument used.

Also of interest in the field of ODD is the good capacity of the ODB trajectories – with all instruments – to predict depression (10% to 27% of the variability of depression was explained by the trajectories). Several studies have associated ODD with emotional symptoms at different ages. For instance, Copeland et al. [30] showed that ODD in adolescence increased the risk of depression in early adult life. Also, Stringaris and Goodman [31] point out that ODD and depression share irritability symptoms, which may account for their predictive association. The present findings indicate that the pattern of prediction may be observable early in life, which entails strong potential for prevention.

Another important finding from this study concerns the relevance of knowing which trajectory a child belongs to, as a useful predictor of outcomes at age 6. To give two examples, pertaining to any of the DSM-IV trajectories discriminated the presence of ODD at a level of 24.7% capacity, and the CBCL-aggressive trajectories explained 22.3% of the group of disruptive disorders at age 6. Depending on the purpose, clinicians and researchers may want to choose one of the instruments, taking into account the specifically-defined trajectories traced. For example, if the objective is to predict comorbidity at age 6, as related to oppositional behaviors, the most predictive definition is that derived from CBCL-aggressive; if the outcome studied is ODD, or impairment, then the solution derived with the DSM-IV-ODD may be the best option.

In summary, our study contributes to knowledge of the field through the identification of several developmental trajectories of oppositional behaviors according to different definitions. It is important to note that the trajectories identified via different instruments tended to concur, and using different definitions, the trajectories summarized longitudinal information on oppositionality in a closer way. However, some discrepancies in the evolution of the ODD problem were observed (not all the trajectories were similar), and this may be inherent to the differences between individual definitions. All definitions could be useful for the study of oppositional behaviors, and all had predictive validity for different outcomes at age 6. However, some limitations should be considered in interpreting these results. First, with the aim of permitting statistical comparison, this study only considered as final GMM with no less than 4% of participants in a class-trajectory, and this may be affecting the results, since this criterion reduces the more finely graded differences between different courses within the ODD spectrum. More research is needed to further test the validity of the resulting trajectories. The questionnaire scales include some items that represent symptoms of CD, but these were a minority in the set, and our objective was to provide clinicians and researchers with the trajectories yielded by the complete scales of the instruments and scores that they routinely use. Also, families with low socioeconomic status participated less than families from other levels, and this could have led to bias. Our results, then, do indeed contribute to the body of knowledge about the development of ODD in preschool children, and suggest future lines of enquiry for research on stability and protective and risk factors for early oppositional-defiant behaviors.

## Supporting Information

Figure S1 **Study design.** SDQ: Strengths and Difficulties Questionnaire; ODD: Oppositional Defiant Symptoms; P3: first year of preschool education (age 3); P4: second year of preschool education (age 4); P5: third year of preschool education (age 5); 1E: first year of elementary school (age 6)
(DOC)

Table S1 **Demographic Characteristics of the Sample (** ***N*** **= 622).**
(DOC)

Table S2 **Fitting Indices for Growth-Mixture-Modeling.**
(DOC)

Table S3 **Comparison between Trajectories on Outcomes at Age 6.**
(DOC)

Table S4 **Item content of the scales used for the trajectories in each instrument.**
(DOC)
