# Supplementary material for: Tracing Developmental Trajectories of Oppositional Defiant Behaviors in Preschool Children
Source: PLoS One. 2014 Jun 27;9(6):e101089. doi: 10.1371/journal.pone.0101089 (PMC4074167; doi:10.1371/journal.pone.0101089)
Supplement: Figure S1 — Study design. SDQ: Strengths and Difficulties Questionnaire; ODD: Oppositional Defiant Symptoms; P3: first year of preschool education (age 3); P4: second year of preschool education (age 4); P5: third year of preschool education (age 5); 1E: first year of elementary school (age 6) (DOC) [file pone.0101089.s001.doc]

1^st^

P

H

A

S

E

2^nd^

P

H

A

S

E

**Screening (−)**

N− = 756

Random sample

n− = 235

**Screening (+)**

Conduct problems ≥ 4

OR any ODD symptom with a score of 2 (certainly true)

N+ = 522 (42.9%)

All

n_1_ = 417

Random sample

N = 2,283

**Census of 3-year-old pre-schoolers**

**in Barcelona (2008)**

N= 13,578 children

**Agree 1341 (58.7%)**

Excluded

12 (0.5%)

Refuse

930 (41.0%)

Excluded

63 (4.6%)

SDQ ^3-4^ Screen

DSM-IV ODD symptoms

**N= 1278 (55.8%)**

Refuse

105 (20.2%)

n_0_ = 205

Refuse

30 (12.8%)

**P3**

**P4**

**P5**

**1E**

***Follow-Up***

Figure S1. Study design.

SDQ: Strengths and Difficulties Questionnaire; ODD: Oppositional Defiant Symptoms; P3: first year of preschool education (age 3); P4: second year of preschool education (age 4); P5: third year of preschool education (age 5); 1E: first year of elementary school (age 6)
