# Supplementary material for: Tracing Developmental Trajectories of Oppositional Defiant Behaviors in Preschool Children
Source: PLoS One. 2014 Jun 27;9(6):e101089. doi: 10.1371/journal.pone.0101089 (PMC4074167; doi:10.1371/journal.pone.0101089)
Supplement: Table S1 — Demographic Characteristics of the Sample ( N = 622). (DOC) [file pone.0101089.s002.doc]

Table S1. Demographic Characteristics of the Sample (*N=*622).

| ***Child*** |  |  | School (n; %) |  |  |
| --- | --- | --- | --- | --- | --- |
| Age (mean; *SD*) | 3.8 | (0.33) | Public | 397 | (63.8) |
| Sex (*N*; %) | 311 | (50.0) | Semi-public | 225 | (36.2) |
| Race/ethnicity (*N*; %) |  |  | Living with… (n; %) |  |  |
| Non-Hispanic white | 557 | (89.5) | Both biological parents | 585 | (94.7) |
| Hispanic- American | 46 | (7.4) | Adoptive parents | 7 | (1.1) |
| Other | 19 | (3.1) | Reconstructed family | 9 | (1,5) |
|  |  |  | One-parent family | 30 | (4.8) |
| Family socioeconomic status (*N*; %) |  |  | Born outside Spain (n; %) |  |  |
| High | 205 | (33.0) | Child | 19 | (3.1) |
| Mean-high | 280 | (45.0) | Mother | 88 | (14.2) |
| Low | 137 | (22.0) | Father | 94 | (15.6) |
| ***Parents*** | ***Mother*** | ***Father*** |  |  |  |
| Age (mean; *SD*) | 36.4 (4.7) | 38.6 (5.8) |  |  |  |
| Education (*N*; %)^1^ |  |  |  |  |  |
| College/university | 340 (54.7) | 279 (45.7) |  |  |  |
| High school/Incomplete college | 178 (28.6) | 197 (32.2) |  |  |  |
| Middle school/Incomplete high school | 92 (14.8) | 122 (20.0) |  |  |  |
| Elementary school or less | 12 (1.9) | 13 (2.1) |  |  |  |
| Occupation (*N*; %) |  |  |  |  |  |
| Major/minor professional/administration | 279 (45.1) | 273 (45.7) |  |  |  |
| Technicians/Clerical/Skilled | 200 (32.3) | 237 (39.6) |  |  |  |
| Unskilled | 140 (22.6) | 88 (14.7) |  |  |  |
| Unemployed | 112 (18.0) | 46 (7.4) |  |  |  |

^1^Education unavailable for 11 fathers.
