# Supplementary material for: Tracing Developmental Trajectories of Oppositional Defiant Behaviors in Preschool Children
Source: PLoS One. 2014 Jun 27;9(6):e101089. doi: 10.1371/journal.pone.0101089 (PMC4074167; doi:10.1371/journal.pone.0101089)
Supplement: Table S2 — Fitting Indices for Growth-Mixture-Modeling. (DOC) [file pone.0101089.s003.doc]

Table S2. Fitting Indices for Growth-Mixture-Modeling.

|  | DSM-IV-ODD; parents | | | | CBCL-aggressive; parents | | | | CBCL-DSM-ODB; parents | | | | SDQ-conduct; parents | | | | SDQ-conduct; teachers | | | |
| --- | --- | --- | --- | --- | --- | --- | --- | --- | --- | --- | --- | --- | --- | --- | --- | --- | --- | --- | --- | --- |
| Num.  trajec. | BIC  (^1^Entr.) | Post- ^2^prob. | Count-size  (^3^weighed) | | BIC  (^1^Entr.) | Post- ^2^prob. | Count-size  (^3^weighed) | | BIC  (^1^Entr.) | Post- ^2^prob. | Count-size  (^3^weighed) | | BIC  (^1^Entr.) | Post- ^2^prob. | Count-size  (^3^weighed) | | BIC  (^1^Entr.) | Post- ^2^prob. | Count-size  (^3^weighed) | |
| 1 | 6107.2 | 1.000 | 622 | (622) | 10279.1 | 1.000 | 618 | (618) | 7335.4 | 1.000 | 617 | (617) | 6502.4 | 1.000 | 622 | (622) | 7129.0 | 1.000 | 620 | (620) |
| 2 | 5880.2 | .918 | 94 | (77) | 10219.8 | .837 | 73 | (57) | 7259.2 | .857 | 73 | (55) | 6420.4 | .852 | 82 | (81) | 6949.0 | .914 | 120 | (119) |
|  | (.904) | .980 | 528 | (545) | (.816) | .961 | 545 | (561) | (.849) | .968 | 544 | (562 | (.807) | .959 | 540 | (541) | (.865) | .976 | 500 | (501) |
| 3 | 5712.2 | .964 | 54 | (43) | 10191.3 | .878 | 25 | (20) | **7244.1** | **.842** | **481** | **(514)** | **6419.9** | **.842** | **490** | **(512)** | 6867.6 | .903 | 46 | (44) |
|  | (.939) | .978 | 116 | (105) | (.794) | .845 | 160 | (145) | **(.831)** | **.924** | **78** | **(58)** | **(.832)** | **.732** | **56** | **(35)** | (.869) | .955 | 435 | (447) |
|  |  | .975 | 452 | (474) |  | .923 | 433 | (454) |  | **.728** | **58** | **(45)** |  | **.922** | **76** | **(75)** |  | .892 | 139 | (128) |
| 4 | **5684.7** | **.910** | **373** | **(417)** | **10188.1** | **.902** | **394** | **(423)** | 7240.9 | .927 | 56 | (41) | 6411.7 | .734 | 49 | (31) | **6794.8** | **.947** | **399** | **(410)** |
|  | **(.919)** | **.936** | **131** | **(107)** | **(.842)** | **.876** | **38** | **(28)** | (.752) | .955 | 183 | (170) | (.814) | .928 | 458 | (488) | **(.917)** | **.948** | **94** | **(84)** |
|  |  | **.973** | **80** | **(73)** |  | **.828** | **159** | **(146)** |  | .869 | 340 | (375) |  | .842 | 111 | (101) |  | **.898** | **68** | **(70)** |
|  |  | **.858** | **38** | **(24)** |  | **.763** | **27** | **(21)** |  | .920 | 38 | (30) |  | .979 | 4 | (2) |  | **.876** | **59** | **(56)** |

^1^Entropy. ^2^On-diagonal posterior probabilities. ^3^In brackets, weighted frequency in each trajectory. Bold: selected solution.
