# Supplementary material for: Tracing Developmental Trajectories of Oppositional Defiant Behaviors in Preschool Children
Source: PLoS One. 2014 Jun 27;9(6):e101089. doi: 10.1371/journal.pone.0101089 (PMC4074167; doi:10.1371/journal.pone.0101089)
Supplement: Table S3 — Comparison between Trajectories on Outcomes at Age 6. (DOC) [file pone.0101089.s004.doc]

## Table S3. Comparison between Trajectories on Outcomes at Age 6.

|  | Results adjusted by other comorbidities at baseline | | | | | |
| --- | --- | --- | --- | --- | --- | --- |
| Trajectory: DSM-IV-ODD-Symptoms | T1(308) | T2(108) | T3(65) | T4(30) | ΔR^2^ | *p* |
| ^1^Disruptive disorders; *%* | 6.77 | 9.87 | 32.44 | 50.48 | **.152** | **<.001** |
| ^1^ADHD; *%* | 5.90 | 7.29 | 15.15 | 13.90 | .028 | .138 |
| ^1^ODD; *%* | 1.54 | 6.54 | 19.15 | 45.03 | **.247** | **<.001** |
| ^1^CD; *%* | 0.05 | -0.08 | 2.01 | 2.83 | **.234** | **<.001** |
| ^1^Depression (major+minor); *%* | 0.07 | 0.60 | 7.13 | 2.70 | **.272** | **<.001** |
| ^1^Anxiety; *%* | 9.00 | 6.60 | 9.29 | 8.14 | .002 | .917 |
| ^1^Comorbidity (more 1 dis.); *%* | 6.52 | 14.06 | 17.57 | 43.82 | **.096** | **<.001** |
| ^1^Use of services; *%* | 12.33 | 19.88 | 30.55 | 32.31 | **.048** | **.005** |
| ^2^Impairment (CGAS: total) | 79.18 | 75.75 | 68.39 | 65.39 | **.152** | **<.001** |
| ^2^CD: Number aggr.symptoms | 0.01 | 0.04 | 0.07 | 0.09 | .014 | .155 |
| ^2^CD: Num non-aggr.sympt. | 0.07 | 0.10 | 0.12 | 0.25 | .013 | .213 |
| Trajectory: CBCL-Aggressive behavior | T1(323) | T2(28) | T3(137) | T4(23) | R^2^ | *p* |
| ^1^Disruptive disorders; *%* | 4.75 | 22.64 | 22.38 | 70.04 | **.223** | **<.001** |
| ^1^ADHD; *%* | 3.42 | 14.45 | 13.34 | 40.40 | **.136** | **<.001** |
| ^1^ODD; *%* | 1.88 | 10.42 | 12.46 | 40.72 | **.198** | **<.001** |
| ^1^CD; *%* | 0.07 | -0.30 | 0.83 | 3.69 | **.184** | **<.001** |
| ^1^Depression (major+minor); *%* | 0.27 | -0.40 | 3.18 | 3.68 | **.134** | **<.001** |
| ^1^Anxiety; *%* | 7.13 | 8.70 | 11.29 | 18.28 | .015 | .196 |
| ^1^Comorbidity (more 1 dis.); *%* | 4.57 | 28.05 | 18.65 | 51.86 | **.175** | **<.001** |
| ^1^Use of services; *%* | 10.38 | 14.00 | 29.91 | 44.48 | **.099** | **<.001** |
| ^2^Impairment (CGAS: total) | 79.39 | 72.13 | 72.16 | 64.27 | **.136** | **<.001** |
| ^2^CD: Number aggr.symptoms | 0.02 | 0.00 | 0.04 | 0.15 | .016 | .063 |
| ^2^CD: Num non-aggr.sympt. | 0.06 | 0.15 | 0.13 | 0.30 | .025 | .075 |
| Trajectory: CBCL-DSM-ODB | T1(396) | T2(64) | T3(51) |  | R^2^ | *p* |
| ^1^Disruptive disorders; *%* | 7.74 | 16.41 | 52.74 |  | **.162** | **<.001** |
| ^1^ADHD; *%* | 5.89 | 10.70 | 21.52 |  | **.044** | **.020** |
| ^1^ODD; *%* | 3.25 | 7.71 | 35.59 |  | **.182** | **<.001** |
| ^1^CD; *%* | 0.03 | 0.00 | 4.67 |  | **.352** | **<.001** |
| ^1^Depression (major+minor); *%* | 0.77 | 0.00 | 6.18 |  | **.101** | **<.001** |
| ^1^Anxiety; *%* | 9.45 | 4.09 | 4.69 |  | .010 | .259 |
| ^1^Comorbidity (more 1 dis.); *%* | 8.32 | 14.17 | 30.14 |  | **.056** | **<.001** |
| ^1^Use of services; *%* | 14.87 | 17.92 | 32.54 |  | **.022** | **.021** |
| ^2^Impairment (CGAS: total) | 78.08 | 73.34 | 67.24 |  | **.058** | **<.001** |
| ^2^CD: Number aggr.symptoms | 0.01 | 0.03 | 0.16 |  | .037 | .109 |
| ^2^CD: Num non-aggr.sympt. | 0.07 | 0.18 | 0.21 |  | **.020** | **.033** |
| Trajectory: SDQ-Conduct-Parents | T1(399) | T2(49) | T3(63) |  | R^2^ | *p* |
| ^1^Disruptive disorders; *%* | 7.56 | 23.55 | 37.31 |  | **.136** | **<.001** |
| ^1^ADHD; *%* | 5.66 | 11.75 | 18.63 |  | **.050** | **.013** |
| ^1^ODD; *%* | 3.37 | 13.55 | 21.84 |  | **.131** | **<.001** |
| ^1^CD; *%* | 0.00 | 0.00 | 3.13 |  | **.340** | **<.001** |
| ^1^Depression (major+minor); *%* | 0.45 | 1.90 | 5.17 |  | **.123** | **.022** |
| ^1^Anxiety; *%* | 8.43 | 13.24 | 7.36 |  | .004 | .544 |
| ^1^Comorbidity (more 1 dis.); *%* | 8.53 | 20.41 | 19.66 |  | **.035** | **.013** |
| ^1^Use of services; *%* | 13.92 | 25.84 | 30.04 |  | **.034** | **.011** |
| ^2^Impairment (CGAS: total) | 78.11 | 72.43 | 69.84 |  | **.043** | **<.001** |
| ^2^CD: Number aggr.symptoms | 0.01 | 0.06 | 0.09 |  | .019 | .136 |
| ^2^CD: Num non-aggr.sympt. | 0.07 | 0.12 | 0.23 |  | **.026** | **.041** |
| Trajectory: SDQ-Conduct-Teachers | T1(333) | T2(71) | T3(57) | T4(50) | R^2^ | *p* |
| ^1^Disruptive disorders; *%* | 5.89 | 15.62 | 26.03 | 34.08 | **.144** | **<.001** |
| ^1^ADHD; *%* | 3.48 | 8.28 | 17.63 | 23.57 | **.129** | **<.001** |
| ^1^ODD; *%* | 3.54 | 8.96 | 8.48 | 18.34 | **.074** | **.003** |
| ^1^CD; *%* | 0.02 | 0.00 | 0.02 | 3.96 | **.364** | **<.001** |
| ^1^Depression (major+minor); *%* | 0.39 | 0.00 | 1.08 | 7.86 | **.207** | **<.001** |
| ^1^Anxiety; *%* | 7.86 | 11.49 | 12.63 | 4.97 | .012 | .499 |
| ^1^Comorbidity (more 1 dis.); *%* | 6.91 | 14.92 | 14.48 | 26.35 | **.065** | **.004** |
| ^1^Use of services; *%* | 12.56 | 22.96 | 21.90 | 30.16 | **.040** | **.013** |
| ^2^Impairment (CGAS: total) | 78.50 | 74.11 | 75.09 | 70.08 | **.033** | **<.001** |
| ^2^CD: Number aggr.symptoms | 0.02 | 0.00 | 0.01 | 0.15 | **.038** | **.033** |
| ^2^CD: Num non-aggr.sympt. | 0.08 | 0.02 | 0.11 | 0.21 | **.021** | **.003** |

*Note* ^1^Proportions at 6 years-old. ^2^Means at 6 years-old. In brackets sample size. Anxiety includes separation, generalized, specific and social phobia.

DSM-IV-ODD-Symptoms: T1: low persistent; T2: decreasers; T3: increasers; T4: high persistent; CBCL-Aggressive behavior: T1: low persistent; T2: decreasers; T3: moderate persistent; T4: high increasers; CBCL-DSM-ODD: T1: low persistent; T2: decreasers; T3: high increasers; SDQ-Conduct-Parents: T1: low persistent; T2: decreasers; T3: high increasers; SDQ-Conduct-Teachers: T1: low persistent; T2: decreasers; T3: increasers; T4: high persistent.

ΔR^2^: change in R^2^ when introducing the trajectories in comparison with the previous step including only covariate.
