# Supplementary material for: Tracing Developmental Trajectories of Oppositional Defiant Behaviors in Preschool Children
Source: PLoS One. 2014 Jun 27;9(6):e101089. doi: 10.1371/journal.pone.0101089 (PMC4074167; doi:10.1371/journal.pone.0101089)
Supplement: Table S4 — Item content of the scales used for the trajectories in each instrument. (DOC) [file pone.0101089.s005.doc]

Table S4 online. Item content of the scales used for the trajectories in each instrument

| DSM-IV | CBCL-aggressive behavior | CBCL DSM-oppositional defiant problems | SDQ-Conduct |
| --- | --- | --- | --- |
| *Item score: 0 - 1*  *Scale range: 0÷ 8* | *Item score: 0 - 1- 2*  *Scale range: 0÷ 36* | *Item score: 0 - 1- 2*  *Scale range: 0÷ 10* | *Item score: 0 - 1- 2*  *Scale range: 0÷ 10* |
| 1. Loses temper  2. Argues with adults  3. Defies rules  4. Annoys people  5. Blames others  6. Touchy, annoyed  7. Angry, resentful  8. Spiteful, vindictive | 1. Argues a lot  2. Mean  3. Demands attention  4. Destroy own things  5. Destroy other’s things  6. Disobedient at home  7. Disobedient at school  8. Gets in fights  9. Attacks people  10. Screams a lot  11. Stubborn, sullen  12. Mood changes  13. Sulks  14. Suspicious  15. Teases a lot  16. Temper  17. Threatens others  18. Loud | 1. Argues  2. Disobedient at home  3. Disobedient at school  4. Stubborn, sullen  5. Temper | 1. Temper tantrums/hot temper  2. Disobedient  3. Fights/bullies  4. Lies or cheats (argumentativeness at age 3)  5.Steals (spitefulness at age 3) |
